# Patient‐derived organoids as a preclinical platform for precision medicine in colorectal cancer

**DOI:** 10.1002/1878-0261.13144

**Published:** 2022-01-01

**Authors:** Young‐Won Cho, Dong‐Wook Min, Hwang‐Phill Kim, Yohan An, Sheehyun Kim, Jeonghwan Youk, Jaeyoung Chun, Jong Pil Im, Sang‐Hyun Song, Young Seok Ju, Sae‐Won Han, Kyu Joo Park, Tae‐You Kim

**Affiliations:** ^1^ Department of Molecular Medicine & Biopharmaceutical Sciences Graduate School of Convergence Science and Technology Seoul National University Korea; ^2^ Cancer Research Institute Seoul National University Korea; ^3^ BioMedical Science and Engineering Interdisciplinary Program (BSEIP) Korea Advanced Institute of Science and Technology Daejeon Korea; ^4^ Department of Internal Medicine Seoul National University Hospital Korea; ^5^ Department of Translational Medicine Seoul National University College of Medicine Korea; ^6^ Graduate School of Medical Science & Engineering (GSMSE) Korea Advanced Institute of Science and Technology Daejeon Korea; ^7^ Department of Internal Medicine and Liver Research Institute Seoul National University College of Medicine Korea; ^8^ Department of Internal Medicine Gangnam Severance Hospital Yonsei University College of Medicine Seoul Korea; ^9^ Department of Surgery Seoul National University College of Medicine Korea

**Keywords:** colorectal cancer, drug repurposing, organoid score, patient‐derived organoids, progression prediction

## Abstract

Patient‐derived organoids are being considered as models that can help guide personalized therapy through *in vitro* anticancer drug response evaluation. However, attempts to quantify *in vitro* drug responses in organoids and compare them with responses in matched patients remain inadequate. In this study, we investigated whether drug responses of organoids correlate with clinical responses of matched patients and disease progression of patients. Organoids were established from 54 patients with colorectal cancer who (except for one patient) did not receive any form of therapy before, and tumor organoids were assessed through whole‐exome sequencing. For comparisons of *in vitro* drug responses in matched patients, we developed an ‘organoid score’ based on the variable anticancer treatment responses observed in organoids. Very interestingly, a higher organoid score was significantly correlated with a lower tumor regression rate after the standard‐of‐care treatment in matched patients. Additionally, we confirmed that patients with a higher organoid score (≥ 2.5) had poorer progression‐free survival compared with those with a lower organoid score (< 2.5). Furthermore, to assess potential drug repurposing using an FDA‐approved drug library, ten tumor organoids derived from patients with disease progression were applied to a simulation platform. Taken together, organoids and organoid scores can facilitate the prediction of anticancer therapy efficacy, and they can be used as a simulation model to determine the next therapeutic options through drug screening. Organoids will be an attractive platform to enable the implementation of personalized therapy for colorectal cancer patients.

## Graphical abstract

Patient‐derived organoids (PDOs) represent valuable *ex vivo* models in evaluating personalized therapeutic options. Here, we demonstrated that organoid score, calculated according to organoid response to drugs prescribed to the matched patient, can be predictive of disease progression in patients with colorectal cancer. We also evaluated additional therapeutic options by *in vitro* drug library screening of FDA‐approved drugs in PDOs. Our data suggest that PDOs could be used for the preclinical evaluation of off‐label therapeutics in patients with cancer.

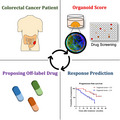

**Abbreviations**
5‐FU: 5‐fluorouracil
AUC: area under drug response curve
CRC: colorectal cancer, Indels, insertions and deletions
MSI‐H: microsatellite instability‐high
PD: progression disease
PDO: patient‐derived organoid
PDX: patient‐derived xenograft
RECIST: response evaluation criteria in solid tumors
SD: stable disease
SNVs: single nucleotide variants
WES: whole‐exome sequencing

## 1 Introduction

Due to the lack of convenient disease models that are genetically representative of heterogeneous cancer tissue, there has recently been increasing interest in stem cell‐based *in vitro* culture systems called organoids [1, 2, 3, 4]. Organoids are three‐dimensional cellular structures cultured *in vitro* from various tissues [5, 6, 7, 8]. Specifically, they are derived from adult stem cells, allowing them to retain the potential to differentiate into multiple lineages *in vitro* [9]. This culture system is more physiologically representative than previous cancer models consisting of a single immortal cell line [10, 11, 12, 13, 14]. Importantly, organoids represent a rapid, flexible, and easily cultivable platform, unlike animal models based on patient‐derived xenografts (PDX) [15]. Furthermore, organoids can be generated from limited amounts of starting materials, such as needle or endoscopic biopsy samples, which allows longitudinal studies of tissue from a single patient [16].

Colorectal cancer (CRC) is the third most common cancer worldwide, and its incidence and mortality continue to increase [17]. Unfortunately, in current practice, patients with CRC, especially those with poor responses to standard‐of‐care therapies, have few therapeutic options. Moreover, responses to currently available therapeutics vary considerably across patients [18, 19]. Patients have varying levels of drug resistance and inter‐ and intra‐tumor heterogeneity, emphasizing the need for personalized therapy [20].

Numerous *in vivo* and *ex vivo* models have been proposed for evaluating potential drug candidates for the personalized therapy of CRC. Patient‐derived two‐dimensional cell lines are common models, but they have several drawbacks. The establishment of primary cell lines from patient tissues is inefficient, and this model carries the risk of failing to capture tumor heterogeneity, which may lead to the false representation of tumor pathology [15]. A PDX model might better capture patient tumor heterogeneity. However, PDX models are challenging because of the long development time, high cost, and possibility of mouse‐specific tumor evolution [8, 15]. Colon cancer organoids have recently been developed as an alternative model for precision medicine. Nevertheless, there have been few studies that have compared drug responses between patients and derivative organoid models [21, 22].

In this study, we successfully derived and cultured organoids from 54 patients with CRC. Using these organoids, we performed drug screening and whole‐exome sequencing (WES). Moreover, we introduced a scoring system called the ‘organoid score’, which reflects the drug responses of patient‐derived organoids (PDOs). The aim of this study was to characterize colorectal tumor organoids, compare clinical drug responses between patients and their matched organoids using the organoid score, and suggest new treatment options for patients with poor clinical outcomes.

## 2 Materials and methods

### 2.1 Human specimens

Human colon tissues were obtained from Seoul National University Hospital (Seoul, Korea). After surgical removal, a portion of each colon tumor and adjacent normal tissue was immediately frozen and stored in liquid nitrogen until use. Other remaining tissues were used to prepare primary cultures. In cases of endoscopic samples, the remaining one or two pieces of samples were used during the tissue pathology diagnosis. Patient clinical data were collected from the medical record system. Tumor size at baseline and after standard‐of‐care therapy was estimated according to Response Evaluation Criteria in Solid Tumors (RECIST). Progression‐free survival data were obtained in accordance with the last visit date or time of disease progression by September 2020. This study was approved by the Institutional Review Board of Seoul National University Hospital (approval number: 1608‐054‐784, 1710‐102‐896). The experiments were undertaken with the understanding and written consent of each subject. The tissue acquisition was performed between August 2017 and September 2019. This study was performed in accordance with the Declaration of Helsinki.

### 2.2 Establishment of patient‐derived organoids

To establish PDOs, normal and tumor tissues were processed as described previously [23, 24] with minor modifications. Briefly, to isolate crypts, adjacent normal mucosa was cut into 1‐ to 2‐mm sections and washed with Dulbecco’s phosphate‐buffered saline (DPBS) until the supernatant was clear. Subsequently, the normal colonic mucosal fragments were gently washed with 2 mm ethylenediaminetetraacetic acid/DPBS chelation buffer and then incubated on ice with shaking. After 1 h, the tubes were shaken vigorously to extract crypts, which were washed using basal medium. The composition of the basal medium was advanced DMEM/F12 (Invitrogen, Carlsbad, CA, USA) supplemented with penicillin/streptomycin (Invitrogen), 10 mm HEPES, and GlutaMAX (Invitrogen). For tumor organoids, fresh tumor tissues were washed with DPBS and minced using a gentleMACS Dissociator (Miltenyi Biotec, Bergisch Gladbach, Germany). Processed samples were passed through a 70‐μm cell strainer to eliminate macroscopic tissue debris and then washed with basal medium. The isolated normal crypts and dissociated tumor cell pellets were seeded with Matrigel (Corning, Corning, NY, USA) in 24‐well plates. After the Matrigel had solidified, organoid medium was added. The composition of the organoid medium was 50% Wnt‐3a or hAFM/Wnt‐3a conditioned medium (for normal organoids only), 10% R‐spondin1 conditioned medium, 10% Noggin conditioned medium or 100 ng·mL^−1^ recombinant Noggin (PeproTech, Cranbury, NJ, USA), 50 ng·mL^−1^ recombinant human EGF (PeproTech), B27 (Invitrogen), 1.25 mm N‐acetyl cysteine (Sigma‐Aldrich, St. Louis, MO, USA), 10 mm nicotinamide (Sigma), 3 μm SB202190 (Sigma), 500 nm A83‐01 (Tocris, Bristol, UK), 10 nm prostaglandin E2 (Sigma), 10 nm gastrin (Sigma), and 100 μg·mL^−1^ Primocin (InvivoGen, San Diego, CA, USA) in basal medium. For normal organoid differentiation, Wnt‐3a conditioned medium was withdrawn in organoid medium. The cell lines for Wnt‐3a/Noggin conditioned medium were kindly provided by H. Clevers. The cell line used to produce hAFM/Wnt‐3a conditioned medium was kindly provided by Prof. Junichi Takagi [25]. The cell line for R‐spondin1 conditioned medium was purchased from Trevigen (Gaithersburg, MD, USA).

For immunohistochemistry, tissues and organoids were fixed in formaldehyde and embedded in paraffin for hematoxylin and eosin (H&E) or CDX2 (1 : 600; BioGenex, Fremont, CA, USA) staining. For whole‐mount immunofluorescence staining, Matrigel‐embedded organoids were carefully collected using organoid harvesting solution (Trevigen). The organoids were fixed and permeabilized in formaldehyde and Triton X‐100, respectively. Primary antibodies against EpCAM (1 : 100; Cell Signaling Technology, Danvers, MA, USA) and F‐actin (1 : 200, Invitrogen) were used for staining, and 4′,6‐diamidino‐2‐phenylindole (Invitrogen) was used for counterstaining.

### 2.3 Organoid culture and passaging

The organoid medium was changed three times per week. For passaging, organoids embedded in Matrigel were collected and dissociated by mechanical disruption. Subsequently, the remaining Matrigel was washed out with cold DPBS and the cells were reseeded with fresh Matrigel. To prepare single‐cell suspensions, organoids were dissociated using TrypLE Express (Invitrogen) before reseeding. All experiments using organoids were processed by organoid passage 15.

### 2.4 Genomic analysis

Genomic DNA was extracted from organoids using a ReliaPrep gDNA Tissue Miniprep System (Promega, Madison, WI, USA). WES was performed using the Illumina HiSeq 2500 platform. Sixteen organoids were sequenced with matched adjacent normal and tumor tissues for mutation concordance analysis. The average coverages were 186‐, 185‐, and 176‐fold for normal tissue, tumor tissue, and tumor organoids, respectively. Paired‐end sequencing reads were aligned to a human reference genome (GRCh37) with bwa‐mem software [26]. Single nucleotide variants (SNVs) and short insertions and deletions (Indels) were identified using mutect2 and strelka2 [27, 28] software, respectively. The initial calls were filtered using in‐house scripts. Mutation signatures were analyzed using mutalisk [29] and mutationalpatterns [30]. Copy‐number variations and structural variations were explored using sequenza and delly2 [31, 32]. The remaining 36 tumor organoids were also sequenced with 75‐fold average coverages. Alignment was performed with the same pipeline. The tumor‐only mode in mutect2 was used for calling SNVs and Indels.

### 2.5 Organoids drug screening

Organoids drug screening was performed as described previously [24] with minor modifications. Briefly, organoids were harvested and dissociated using TrypLE Express. Then, organoids were resuspended in 2% Matrigel/organoid medium. Approximately 1000–1500 cells per well were seeded in 96‐well plates and treated with various chemotherapeutic drugs. A cell viability assay was performed using CellTiter‐Glo 3D (Promega) at 6 days after drug treatment. To screen the National Cancer Institute (NCI) drug library, compounds were dispensed and diluted using an automatic liquid handler in a 6‐point, 10‐fold serial dilution from 10 µm. To estimate the drug responses of the Matrigel‐embedded organoids, cells were seeded in 24‐ or 48‐well plates and then treated with drugs at 2 days after seeding. The drug library was provided by the US NCI (approved oncology drugs set, DTP compound order number: 31684).

### 2.6 Organoid score

The area under the drug response curve (AUC) was normalized by the maximum AUC for each of four drugs (5‐fluorouracil [5‐FU], oxaliplatin, SN38, and cetuximab). Then, PDOs were divided into four groups normalized by AUC quartiles, and a drug score was assigned to each group (Group 1 = 1, Group 2 = 2, Group 3 = 3, and Group 4 = 4). In accordance with the drug score, the organoid score was calculated for the standard‐of‐care regimen for the particular patient using the following equation;

$$\text{Organoid}\;\text{score}=\sum_{1}^{n}\text{Drug}\;\#1\;\text{score},\;\text{Drug}\;\#2\;\text{score}\;\ldots \;\text{Drug}\;\#n\;\text{score}/n,$$

*n* = number of drugs the patient received in clinic.

For example, when patient 001 received 5‐FU‐ and oxaliplatin‐based combination therapy (e.g., FOLFOX), only drug scores for 5‐FU and oxaliplatin were used to calculate the organoid score of patient 001‐derived organoid.

### 2.7 Statistical analysis

Survival analyses were performed using the Kaplan–Meier method. The differences in progression‐free survival between the groups according to the organoid score were compared using the log‐rank test. Hazard ratios (HRs) were estimated using the Mantel–Haenszel test. The correlation between tumor size changes and organoid scores was analyzed using Spearman’s rank correlation coefficient. All statistical analyses were performed using graphpad prism 8 (San Diego, CA, USA).

## 3 Results

### 3.1 Organoids recapitulate the morphological features of the original tumor

**Fig. 1:**
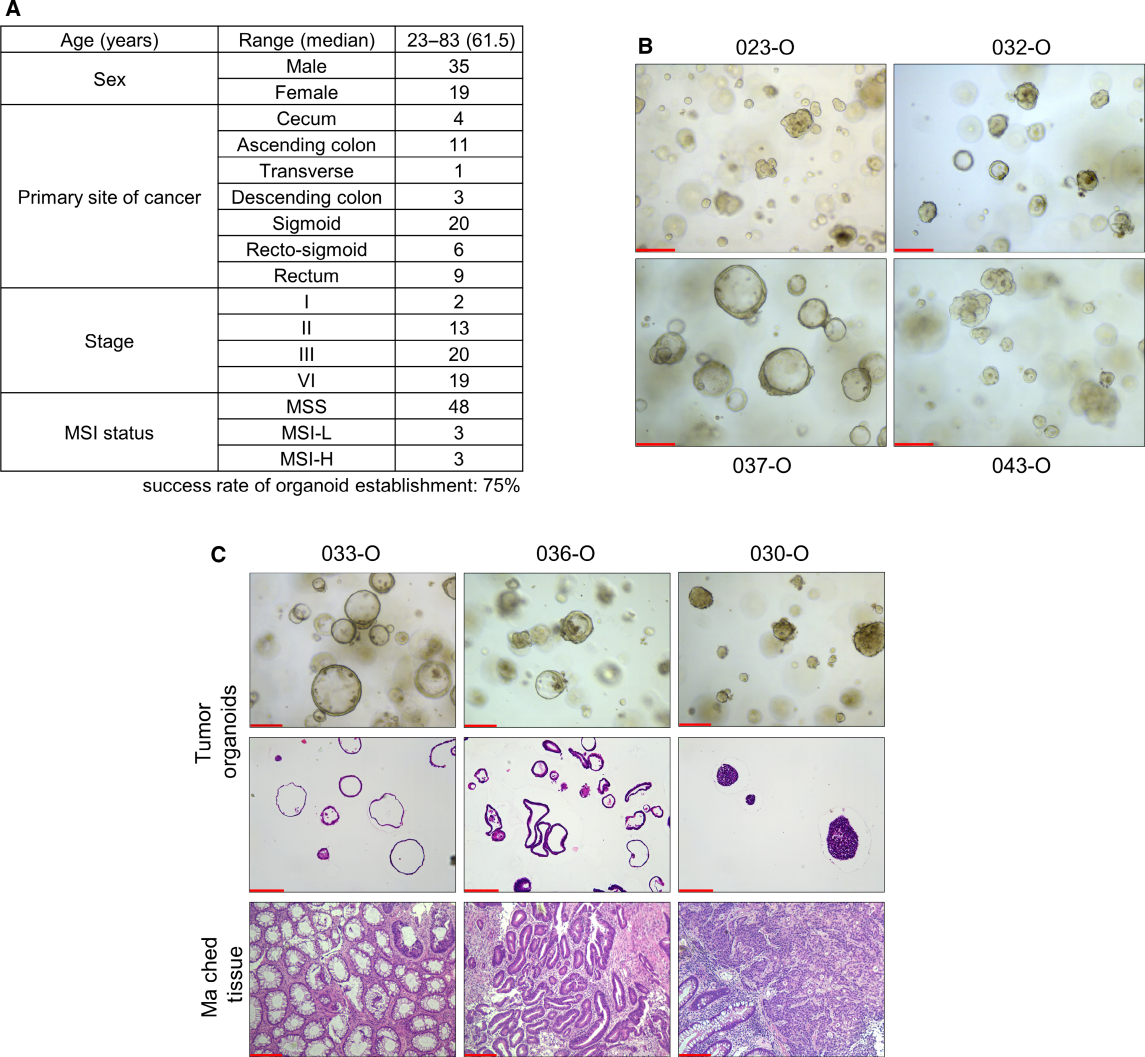
Establishment of organoids from patients with colorectal cancer. (A) Summary of patient information in this tumor organoid study. Organoids were derived from variable colon and rectum location with 75% of success rate. (B) Representative images of tumor organoid morphology derived from different patients. PDOs exhibited varying morphology, namely a cystic (e.g., 037‐O, *n* = 3), aggregated (e.g., 043‐O, *n* = 3), or mixed form (e.g., 032‐O, *n* = 4). Scale bar = 200 µm. (C) Representative images of PDOs compared with H&E‐stained matched original tumor tissues and organoids (*n* = 2). The cultured PDOs and matched tissues showed similar morphology. Scale bar = 200 µm. H&E, hematoxylin and eosin; MSI‐H, microsatellite instability‐high; MSI‐L, microsatellite instability‐low; MSS, microsatellite stability; PDOs, patient‐derived organoids.

To derive PDO, freshly resected colonic adjacent normal and tumor tissues from surgical and endoscopic samples were used. Then, variable anticancer drug responses were tested and compared with patient responses in the clinic (Fig. S1). Tissues from 54 patients, who mostly had stage III or IV CRC, were collected for culture (Fig. 1A, Table S1). Additionally, 53 of 54 patients in this cohort did not receive any prior treatment before tissue collection, and tissues were collected from diverse locations of the colon with varying levels of microsatellite stability. The success rate of tumor organoid establishment was approximately 75%. Organoid culture was considered successful when the cells were sufficiently abundant to be cryopreserved by the second or third passage. The two main causes of failure were a lack of growth and fibroblast overgrowth.

Organoids are often defined as clusters of epithelial cells grown from tissue‐derived adult stem cells or cancer cells via self‐organization [33]. In addition, various tumor organoid culture methods have been developed from the culture methodology of normal adult stem cell organoids [10]. Thus, to check our organoid culture conditions, we investigated whether the characteristics of various colon normal organoids were retained. Because of various existing mutations, tumor organoids had limited ability to display well‐known organoid characteristics such as differentiation or expression signature compared with normal organoids. Through immunostaining, we confirmed that normal organoids expressed an epithelial cell marker (EpCAM) and colon marker (CDX2) and developed a single‐layered structure (Fig. S2A,B) [34]. Under culture conditions without Wnt‐3a conditioned medium, most normal organoids exhibited disruption of the single‐layered organoid structure (Fig. S2C). Through RNA sequencing, we also confirmed that our colon normal organoids displayed an EPHB2‐high human colon stem cell signature [35] compared with differentiation conditions as reported previously (Fig. S2D). Furthermore, changes in the RNA expression of variable stem cell markers (*EPHB2*, *LGR5*, and *ASCL2*) and differentiation markers (*MUC2*, *CHGA*, and *KRT20*), which depended on normal or differentiation medium, were confirmed by qRT‐PCR (Fig. S2E).

Tumor organoids were grown without Wnt‐3a conditioned medium to eliminate normal colon cell contamination in tumor tissues. During growing, organoids exhibited diverse morphologies including cystic structures, compact structures, or a mixture of the two (Fig. 1B). For example, the mixed morphological feature of patient 032‐derived tumor organoids (hereafter shortened to ‘032‐O’) was maintained after more than 6 months of culture, cryopreservation, and single‐cell dissociation (Fig. S3). H&E staining was performed for tumor organoid and matched tissues that were frozen at the time of primary culture (Fig. 1C). The histology of cultured 033‐O and matched tissue included a round and cystic shape with an empty lumen. Conversely, 030‐O and matched tissue exhibited a compact and aggregated structure without an empty lumen. We also confirmed that 036‐O and matched tissue had a cystic and lumen structure but a thicker monolayer structure than 033‐O. These results indicate that our cultured organoids reflect the original tissue morphology.

### 3.2 Genetic comparison of organoids and matched tissue

To assess the genetic alteration profile, WES was performed using 54 patient‐derived tumor organoids (Table S2). For a more detailed approach, 16 tumor organoids were sequenced in parallel with matched normal and tumor tissues.

**Fig. 2:**
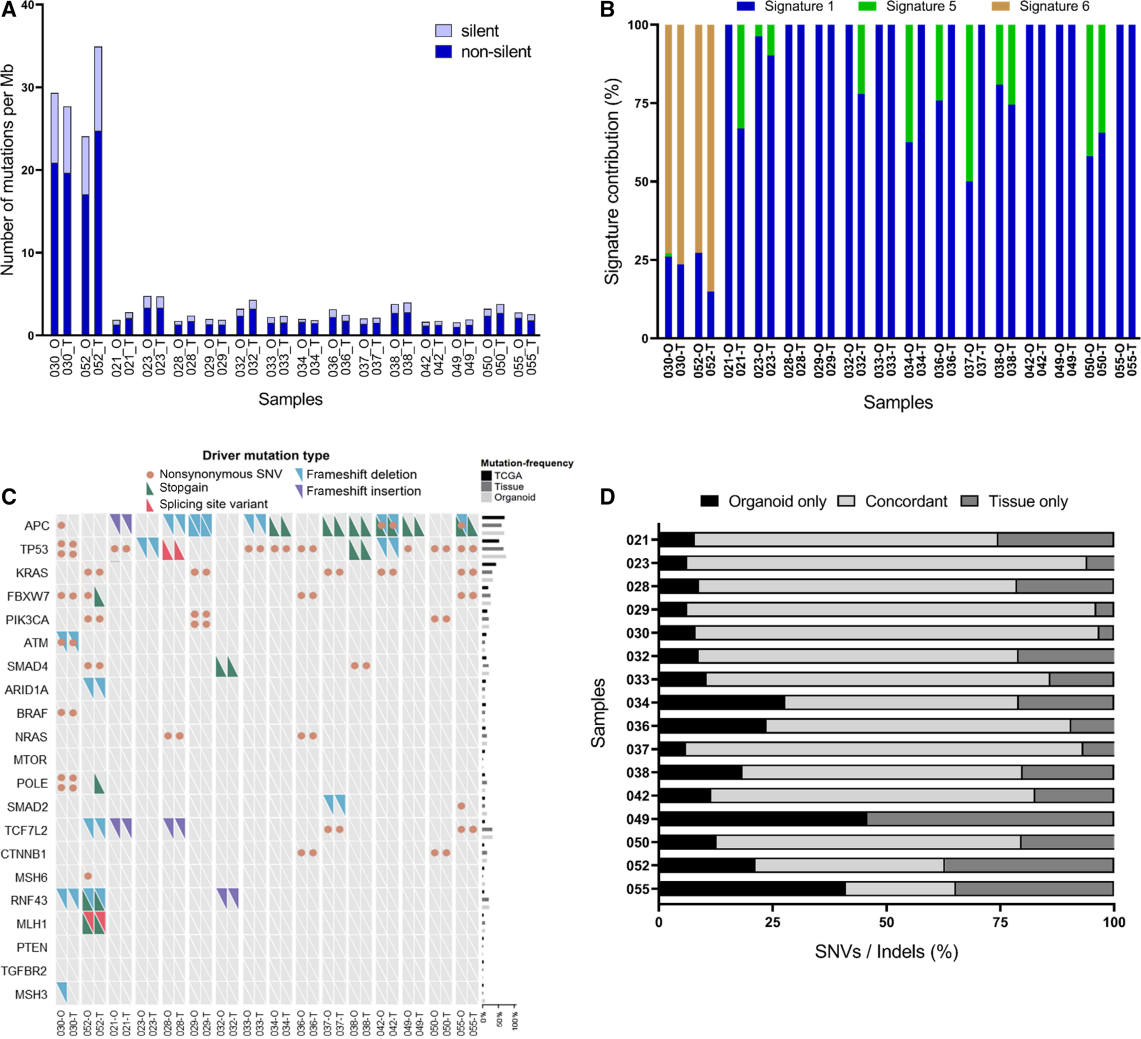
Mutation analysis of PDOs and matched tumor tissues. Sixteen organoids were used as representative analysis for comparisons with tumor tissues, and normal tissues for germline controls. (A) Silent and nonsilent mutation rates per megabase were compared between organoids and matched tissues. Patients 052 and 030 (MSI‐H) possessed relatively higher mutation rates than the other patients. (B) Mutation signatures were analyzed. MSI‐H PDOs and matched tissues primarily exhibited “signature 6”, whereas other samples displayed different signatures. (C) Oncogrid of the colorectal cancer driver gene status of organoids and matched tissues. Organoids and matched tissues showed concordance for 96% in driver gene mutations including *WNT* pathway‐related gene. (Right bar graph) Mutation frequency of tumor organoids and matched tissues were compared with colorectal cancer data in TCGA. Organoids and TCGA cohorts show similar mutation frequencies. (D) Concordance of coding region alterations detected in organoids and matched tissues. The relative proportion of alterations are presented as a bar graph. Median overall concordance of SNV and Indels was 70.08%. Indels, insertions and deletions; MSI‐H, microsatellite instability‐high; PDOs, patient‐derived organoids; SNV, single nucleotide variant; TCGA, the cancer genome atlas.

First, the number of mutations per megabase (Mb) was analyzed, which included the proportions of silent and nonsilent mutations (Fig. 2A). The number of mutations per Mb in non‐hypermutated samples ranged from 1.58 to 4.79 per Mb (median = 2.38 per Mb). Two patient samples were considered to be hypermutated because they had more than 10 mutations per Mb (median = 28.51 per Mb, ranging from 24.06 to 34.92 per Mb). The two hypermutated samples originated from tumor patients with microsatellite instability‐high (MSI‐H). By sequencing analysis, we confirmed that these two MSI‐H cancer tissues had mutations in genes related to DNA repair, included *MLH1* and *POLE*, in both the original tissue and cultured organoids.

In general, the dominant mutational signatures were highly similar in cultured organoids and the matched patient tumor tissues (Fig. 2B). The C‐to‐T transition was the most common mutation, and its relative proportion was similar between matched colon cancer tissue and tumor organoids. Moreover, these results were consistent with previous findings [11]. For example, 023‐O and 033‐O dominantly possessed mutational signature 1. 030‐O and 052‐O, which were derived from patients with MSI‐H tumors, primarily exhibited mutational signature 6, which is frequently observed in MSI‐H tumor samples. These results suggested that the organoid samples recapitulated numerous aspects of the mutational profiles, such as the base transition and mutational signature patterns of tumor tissues.

Frequently mutated and known driver genes of CRC were simultaneously detected in both organoids and tissues (Fig. 2C). Overall, we confirmed that organoids and matched tissues exhibited concordance for 96% in driver gene mutations. Importantly, genes related to the *WNT* signaling pathway were mutated in all analyzed tissues and organoids. *APC* was mutated in 12 of 16 organoids. Conversely, *APC* wild‐type organoids also harbored other *WNT* signaling pathway‐related gene mutations known to be mutually exclusive with *APC*, such as *CTNNB1, RNF43,* and *ARID1A*. These results were consistent with previous findings [36, 37] that *WNT* pathway activation is critical and essential for the development of CRC. Additionally, a manual check was performed for 21 major driver genes. As a result, a homozygous large deletion of *RNF43* was found in 023‐O and matched tumor tissues. Additionally, patient 032 possessed a homozygous large deletion on *TP53* in both tumor organoids and tissues (Fig. S4). To explore whether the mutations detected in our cohort were in line with the mutation profiles generally observed in patients with colon cancer, we compared our results with data from The Cancer Genome Atlas (TCGA) (Fig. 2C; right bar graph). The mutation frequency detected in our samples was consistent with TCGA data, which revealed frequent mutations in major genes such as *APC*, *TP53*, and *KRAS* and relatively infrequent mutations in critical oncogenes such as *PIK3CA*, *NRAS*, and *BRAF*. This indicated that realistic inter‐tumor heterogeneity was reflected in our cohort of cultured organoids and tissues.

We also analyzed the overall concordance of single nucleotide variants (SNVs) and insertions and deletions (Indels) between PDOs and matched tumor tissues (Fig. 2D). Most samples shared numerous mutations between organoids and tumor tissues (median = 70.08% frequency of concordance, ranging from 41.56% to 89.84%). However, samples from patients 049 and 055 had low concordance, and they were thus excluded from subsequent analysis. To ensure that all organoids were derived from matched tumor tissue, WES data were analyzed using NGSCheckMate [38], and we confirmed the absence of mismatched samples (Fig. S5). These variable mutation analyses demonstrated that most organoids were similar to the original tumor regarding the mutation signature, pattern, and type.

### 3.3 Four major drug responses in CRC patient‐derived organoids

**Fig. 3:**
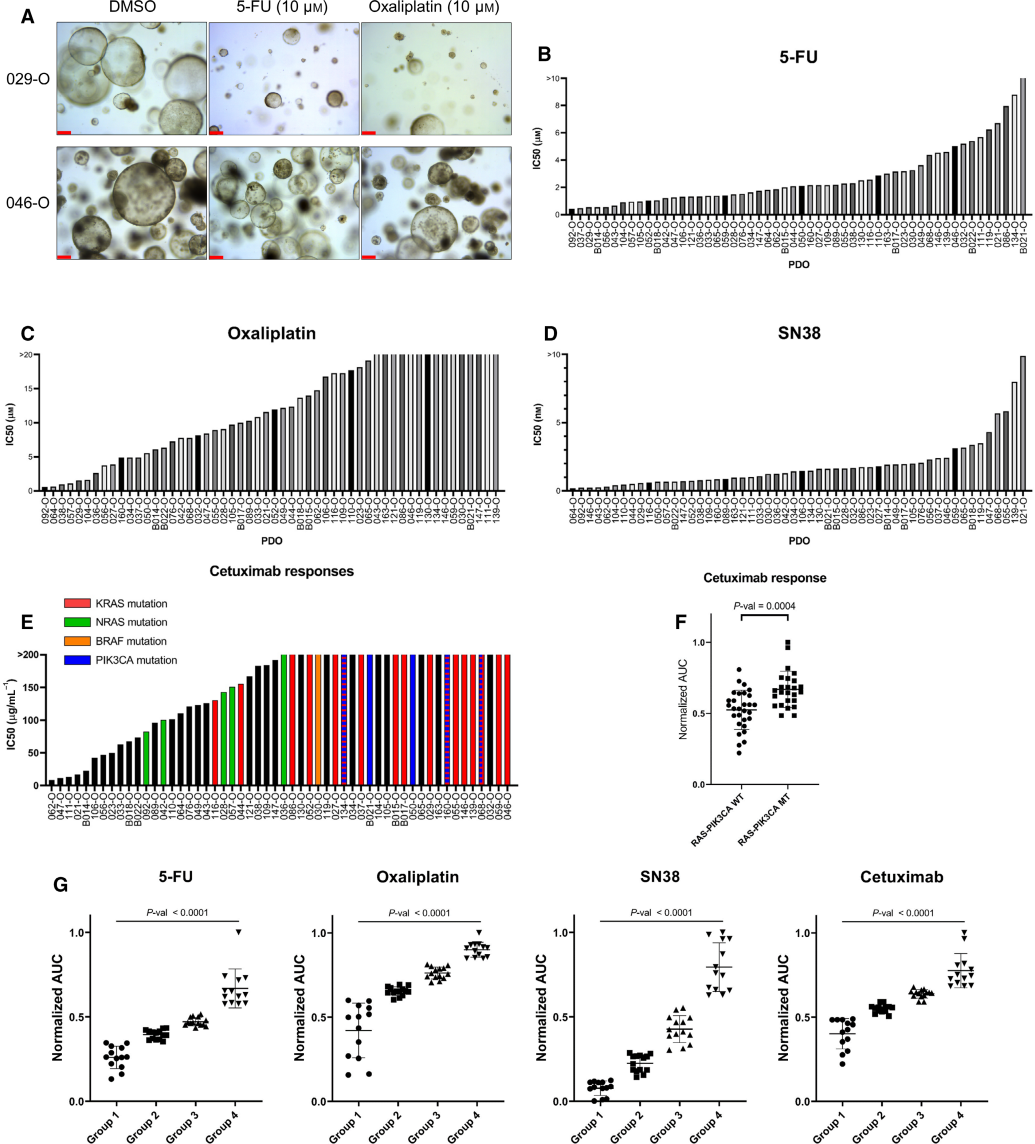
Clinical‐level anticancer drug responses in PDOs. (A) Representative images of differential growth inhibition by chemotherapeutic drugs using chemotherapy‐sensitive (029‐O) or chemotherapy‐resistant (046‐O) organoids. Of note, the sample from patient 046 was obtained after multiple lines of chemotherapy. The remaining samples were obtained before any form of treatment. Images were obtained 6 days after drug treatment. Scale bar = 200 µm. (B–D) IC_50_ bar graph of 5‐FU (B), oxaliplatin (C) and SN38 (D) in PDOs (*n* = 3). (E, F) IC_50_ bar graph (E, *n* = 3) and normalized area under the drug response curve dot plot (F) of cetuximab in organoids with or without *KRAS*, *NRAS* (G12, G13, Q61), *BRAF* (V600E), and *PIK3CA* (E545K, H1047R) hotspot mutations. Tumor organoids harboring mutations exhibited relatively resistance to cetuximab. *P*‐value was estimated using the Mann–Whitney test. Data are expressed as the mean ± SD. (G) Groupings of 54 PDOs based on the normalized area under the drug response curve. A different drug score was assigned for each response group for each drug. *P*‐values were estimated using the Kruskal–Wallis test. Data are expressed as the mean ± SD. 5‐FU, 5‐fluorouracil; PDOs, patient‐derived organoids.

To compare the effectiveness of anticancer drugs, we cultured organoids in the presence of chemotherapeutic drugs commonly prescribed to patients with CRC and measured the anticancer effect on cell viability. Utilizing the 3D organoid culture system, we also confirmed *in vitro* phenotypic changes after drug treatment in several organoids (Fig. 3A). As a result, we found that 029‐O showed relatively poor organoid formation and growth when 5‐fluorouracil (5‐FU) and oxaliplatin were added to the culture medium. Conversely, 046‐O, which was isolated from a patient who did not respond to multiple lines of therapies, was unaffected by high concentration of 5‐FU and oxaliplatin. Surprisingly, the anticancer drugs exhibited a range of IC_50_ values when added to the culture medium rather than producing a dichotomous effect (e.g., either mostly killed or mostly alive; Fig. 3B–D). This implied that the established PDOs possessed a greater degree of inter‐tumor heterogeneity concerning their resilience against drugs than expected.

Cetuximab is prescribed limitedly for patients with CRC. One of the important factors in selecting an anticancer therapy regimen that includes cetuximab is the presence of mutation. We cross‐checked the responses to cetuximab and tumor organoid mutations in the WES results. Tumor organoids harboring *KRAS*, *NRAS*, *BRAF,* or *PIK3CA* hotspot mutations exhibited a relatively higher IC_50_ and area under the drug response curve (AUC) for cetuximab than organoids without hotspot mutations in these genes (Fig. 3E,F). These results indicated that the various patient‐derived tumor organoids possessed heterogeneous anticancer drug responses. Next, we examined whether these drug responses in PDOs were similar to those in the matched patient.

### 3.4 Organoid drug response comparisons with patient clinical responses

First, to determine whether PDOs successfully mimicked the patient response to therapy for use as a co‐clinical model, we compared the *in vitro* drug responses and matched patient responses for each drug (Table 1). Every tumor sample except that from patient 046 was collected before the patient received any form of therapy. The IC_50_ of 5‐FU was < 1.5 μm in B014‐O and 028‐O (Fig. 3B, Table S3), and this value was lower than the maximum serum concentration of 5‐FU [39]. Of interest, patients B014 and 028 received 5‐FU‐based therapy and showed partial response (PR) in the clinic (Table 1, Table S1). Conversely, in the case of patient 032, the tumor did not show a meaningful regression even after treatment with both 5‐FU‐based oxaliplatin combination therapy (FOLFOX) and irinotecan combination therapy (FOLFIRI). *In vitro*, 032‐O also exhibited relative resistance to 5‐FU, oxaliplatin, and SN38 (Table 1, Fig. 3B–D). 046‐O was derived from ascites, and at the time of sample collection, tumor progression had not been slowed despite treatment with multiple lines of anticancer regimens including FOLFOX and FOLFIRI. In concordance with the clinical history of patient 046, this ascites‐derived organoid was relatively resistant to various chemotherapeutic agents in organoid culture. In the case of patient 021, 021‐O was virtually unaffected by 5‐FU, displaying the fourth highest 5‐FU IC_50_ out of all 54 tested organoids (IC_50_ = 6.7 μm). However, the IC_50_ of cetuximab in 021‐O was the fourth lowest among the tested tumor organoid samples (Fig. 3E). Patient 021 received multiple cycles of cetuximab with FOLFOX combination therapy and showed a PR to this regimen (Table 1). Throughout seven cycles of cetuximab monotherapy, the patient continued to show a PR. Of interest, in both organoids and matched tissues from patient 021, *APC*, *TP53*, and *TCF7L2* gene mutations were identified, but no mutations were detected in the MAPK pathway (Fig. 2C).

**Table 1:** *In vitro* PDO drug responses and matched patient responses. PFS, progression‐free survival; PR, partial response; SD, stable disease.

Patient number	# B014	# 028	# 032	# 046	# 021
IC50 in PDO (sensitivity rank in this PDO cohort, *n* = 54)					
5‐FU (μm)	0.55 (4)	1.49 (20)	5.20 (47)	5.03 (46)	6.70 (51)
Oxaliplatin (μm)	6.11 (14)	9.06 (22)	8.13 (19)	37.07 (44)	11.57 (27)
SN38 (nm)	1.93 (38)	1.64 (33)	1.68 (34)	2.42 (45)	9.88 (54)
Cetuximab (μg·mL^−1^)	22.35 (5)	143.1 (21)	7.92E+56 (52)	1.14E+63 (54)	16.91 (4)
Response in patient					
Regimen	FOLFIRI + Bevacizumab	FOLFIRI + Cetuximab	FOLFOX + Bevacizumab	Multiple line of therapy^a^	FOLFOX + Cetuximab
Best response	PR	PR	SD	N/A^b^	PR
PFS (days)	393	413	116		612

^a^ FOLFIRI, FOLFOX, cetuximab + irinotecan, Xeloda, regorafenib.

^b^ Patient 046‐derived ascites was the only one samples, obtained after multiple line of therapy.

### 3.5 Correlation of the organoid score and therapy response or progression in patients

**Fig. 4:**
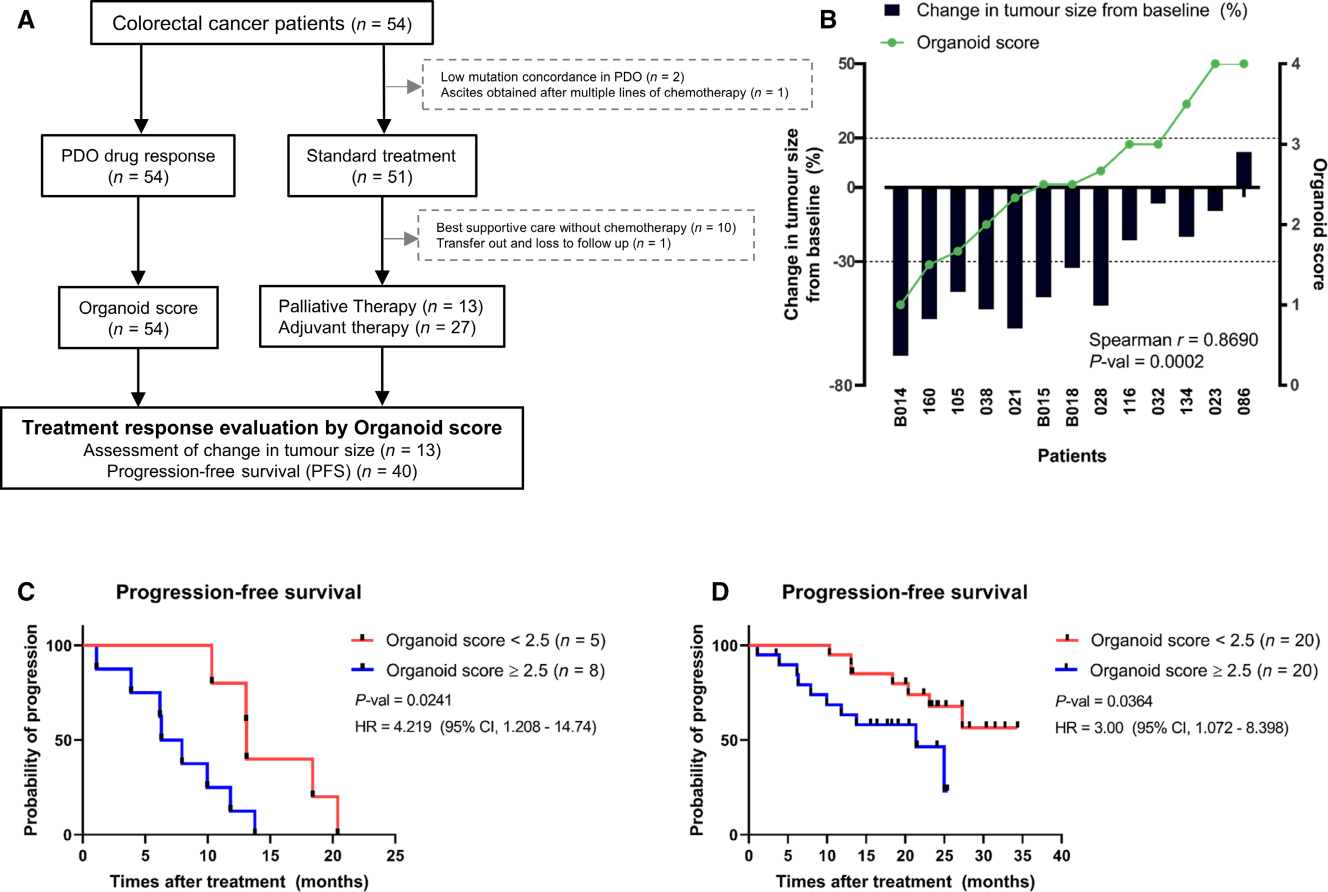
Comparison of *in vitro* drug responses and clinical outcomes. (A) Flowchart of the number of PDOs, evaluable patients, and reasons for dropout. Of the 54 patients, 14 patients were excluded from this analysis. (B) Correlation of organoid scores and tumor size changes in matched patients (*n* = 13). High organoid scores were correlated with low tumor regression. *P*‐value was estimated using Spearman’s rank correlation coefficient test. (C, D) Kaplan–Meier analysis using the organoid score in palliative therapy group (*n* = 13) (C) and overall group (*n* = 40) (D). High organoid score (≥ 2.5) group had worse prognosis than those with low organoid score (< 2.5) group. *P*‐values were estimated using the Mantel–Cox log‐rank test. The HRs were estimated using the Mantel–Haenszel test. CI, confidence interval; HR, hazard ratio; PDOs, patient‐derived organoids.

Next, to explore the utility of PDOs as a prognosis prediction model at the cohort level, drug responses in organoids were compared with the response to standard‐of‐care therapy and disease progression in patients. To accomplish this approach, we developed a scoring system called the ‘organoid score’, which was calculated according to organoid drug responses to particular drugs that were prescribed to the matched patient. 54 PDOs were divided into four response groups for each drug (Fig. 3G) and assigned the drug score. Then, the organoid score was calculated to reflect the drugs received by the matched patient (more details provided in the Methods). Of the 54 patients, 10 patients (patients 030, 052, 059, 092, 104, 109, 146, 147, B017, and B022) were excluded from this analysis because they received best supportive care without chemotherapy. Four patients were additionally excluded because of the following reasons: low mutation concordance between PDO and matched tissue (*n* = 2; patients 049 and 055), ascites obtained after multiple lines of chemotherapies (*n* = 1; patient 046), and transfer and loss to follow‐up (*n* = 1; patient 163; Fig. 4A, Table S1).

In the palliative therapy group, tumor sizes were measured in accordance with the RECIST criteria in patients. Then, the rate of the tumor size change after first‐line therapy was compared with the organoid scores from matched PDOs. Very interestingly, we confirmed that high organoid scores in PDOs were significantly correlated with a change in the tumor size from baseline to after standard‐of‐care treatment in patients (Spearman’s *r* = 0.8690, *P*‐val = 0.0002; Fig. 4B). In the case of patient 023, the organoid score for the FOLFOX regimen was 4, which was the highest score. The observed response to first‐line therapy in patient 023 was stable disease (SD), but the patient exhibited early progression after 6 months of first‐line treatment.

For an overall comparison of *in vitro* organoid scores and patient progression, we also performed Kaplan–Meier analyses using the organoid score and progression‐free survival data of patients (Fig. 4C,D). In the palliative therapy group, we confirmed that patients with organoid scores of 2.5 or higher had a significantly worse prognosis than those with organoid scores of lower than 2.5 (*n* = 13, *P*‐val = 0.0241). In the overall group, we also observed a worse prognosis in patients with higher organoid scores than those with lower organoid scores (*n* = 40, *P*‐val = 0.0364). Through comparing organoid scores with standard‐of‐care responses in matched patients, we confirmed that the PDOs were generally representative of patient responses to standard therapeutic drugs. In addition, drug responses in PDOs could predict progression in patients.

### 3.6 Differential responses of RNF43‐mutated organoids and the implications for precision medicine

**Fig. 5:**
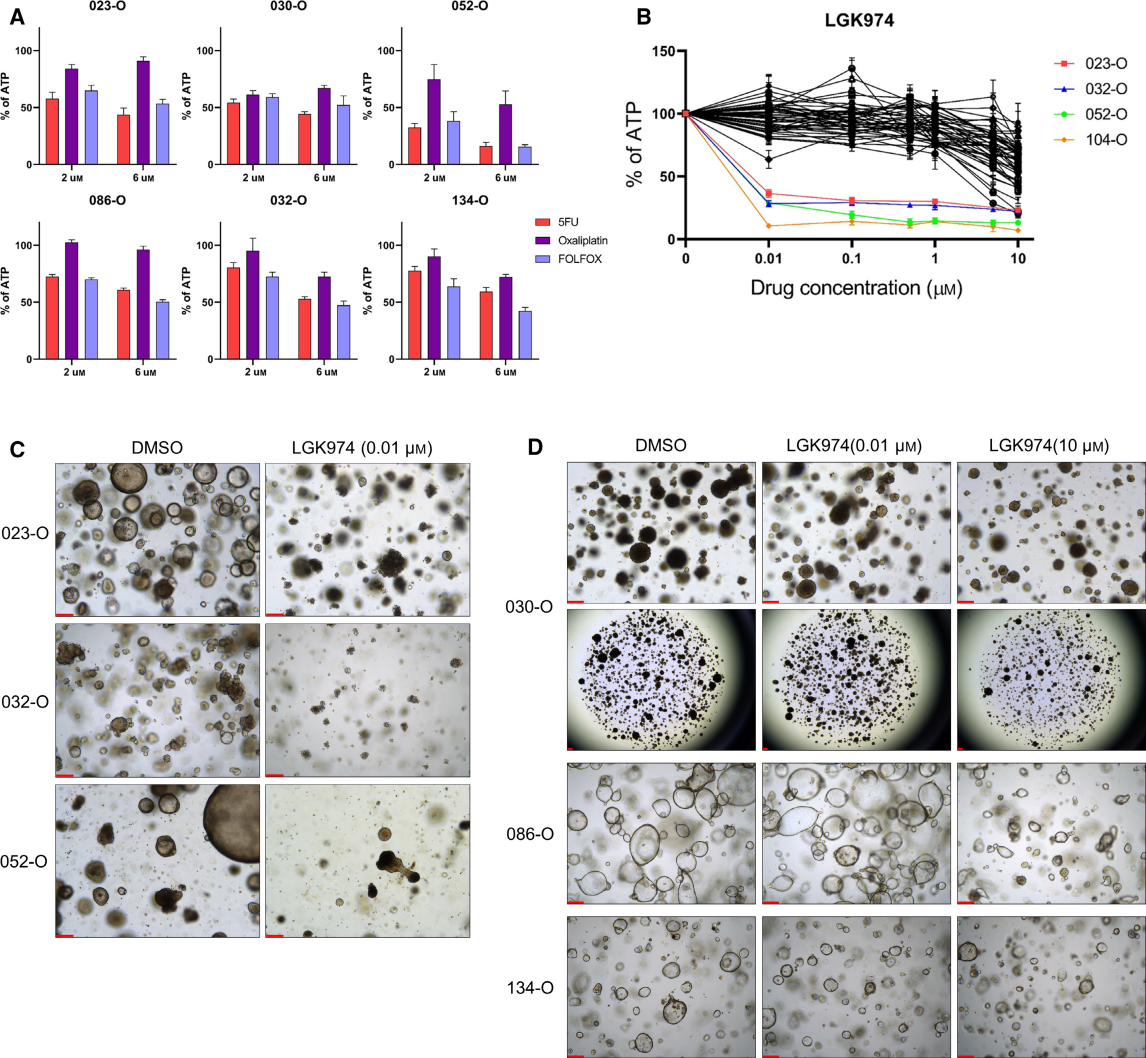
Porcupine inhibitor is a promising anticancer drug for treatment of PDOs harboring *RNF43* mutations. (A) Representative drug responses of six PDOs to single or combination treatment with 5‐FU and oxaliplatin. Data are expressed as the mean ± SD (*n* = 3). *Y*‐axis is the normalized ATP level relative to DMSO. (B) Response curve for the porcupine inhibitor LGK974. (Colored line) 023‐O, 032‐O, 052‐O, and 104‐O, which commonly harbored *RNF43* mutation, displayed hypersensitivity to LGK974. Data are expressed as the mean ± SD (*n* = 3). *Y*‐axis is the normalized ATP level relative to DMSO. (C) Representative images of organoid formation and growth inhibition after LGK974 treatment in *RNF43‐*mutated PDOs. Images were obtained 6 days after treatment with 10 nm LGK974 (*n* = 3). Scale bar = 200 µm. (D) 030‐O was resistant to LGK974 despite carrying an *RNF43* mutation, similar to the *RNF43* wild‐type 086‐O and 134‐O. Images were obtained 6 days after 0.01 or 10 μm drug treatment (*n* = 3). Scale bar = 200 µm. 5‐FU, 5‐fluorouracil; PDOs, patient‐derived organoids.

For patients with tumors that were resistant to first‐line therapy, we investigated whether PDOs could be used as preclinical models to identify alternative treatment options. As described previously, patient 023 received FOLFOX therapy, but disease progression occurred 6 months after first‐line treatment. *In vitro* testing of 023‐O also did not reveal sensitive responses (organoid score = 4, Fig. 4B). To confirm this result, organoids were treated with a combination of 5‐FU and oxaliplatin at concentration approximating the mean and maximum plasma levels of the drugs (Fig. 5A). However, there was no discernible advantage to combining these drugs compared with the effect of 5‐FU alone *in vitro*. In five representative PDOs, the anticancer effect of 5‐FU and oxaliplatin co‐treatment was also not remarkable.

To identify potential alternate anticancer drug treatments, we screened for druggable targets based on WES data (Fig. 2C, Table S4). Patient 023‐derived organoids and tissue harbored an *RNF43* gene mutation, the only well‐known mutation related to the *WNT* pathway. The mutation in *RNF43*, a frizzled E3 ligase, is predictive of a positive response to porcupine (PORCN) inhibitor treatment [40]. On the basis of this information, LGK974, a PORCN inhibitor currently being investigated in clinical trials (NCT01351103), was screened in established PDOs, including 023‐O (Fig. 5B). Consequently, 023‐O, 032‐O, 052‐O, and 104‐O, which were four of the five PDOs harboring an *RNF43* gene mutation, displayed dramatic reductions in sizes and growth and extremely low IC_50_ values for LGK974 (IC_50_ = 9.2, 7.2, 6.6, and 4.0 nm, respectively; Fig. 5C, Fig. S6). However, 030‐O, which also possessed an *RNF43* mutation, did not respond to treatment with LGK974 (IC_50_ = 6.2 μm), similarly as *RNF43* wild‐type organoids including 086‐O and 134‐O (IC_50_ = 25.7 and 23.7 μm, respectively), compared with sensitive organoids (Fig. 5D). Of note, 030‐O harbored a minor point mutation in *APC* (c.T4341A) with a low mutated allele frequency (5%). Conversely, a frameshift deletion in *RNF43* (c.1595delG) exhibited a high mutated allele frequency (90%). It could be considered that driver mutation for *WNT* pathway activation might be present *RNF43* rather than *APC*. Nevertheless, other strategies are needed to achieve tumor regression in 030‐O.

### 3.7 Drug‐repurposing strategies based on drug screening in PDOs

**Fig. 6:**
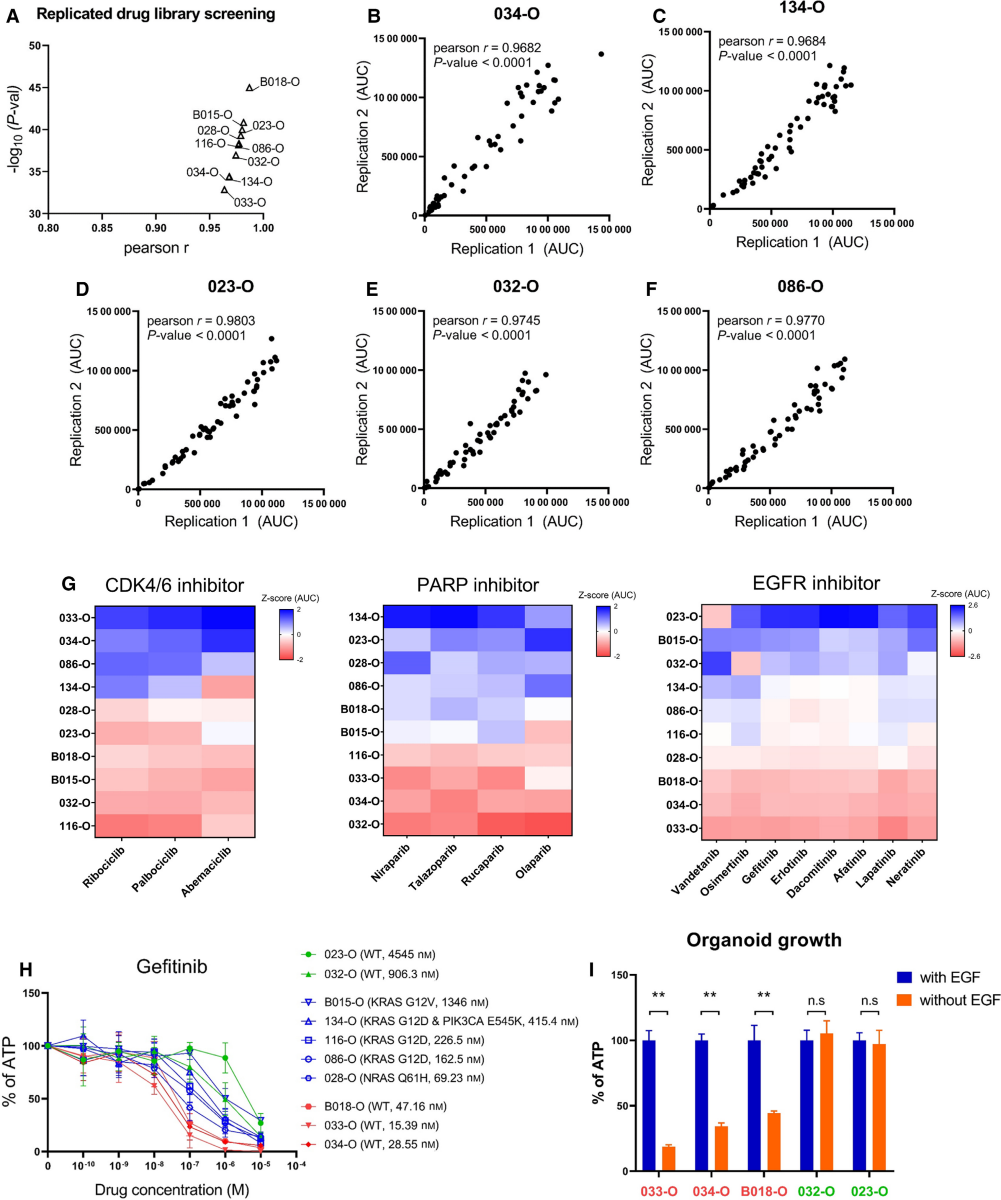
Screening of 57 FDA‐approved anticancer drugs for the treatment of standard therapy‐refractory PDOs. (A) Correlation of two independent drug screening results in 10 PDOs (median Pearson’s *r* = 0.978). (B–F) Representative data of replicated drug screening results (*n* = 2). Pearson’s *r* and *P*‐values were estimated using the Pearson correlation coefficient test. (G) Representative data of a similar response trend to molecular targeted drugs that share the same target in PDOs (*n* = 2). Three or four drugs that targeted CDK4/6 and PARP and eight drugs that targeted EGFR/HER2 produced similar responses in PDOs. The *Z*‐score was calculated using the area under the drug response curve, indicating the relative treatment efficiency. (H) Drug response curves for gefitinib in various PDOs in accordance with the presence of *KRAS*, *NRAS*, and *PIK3CA* oncogenic mutation (*n* = 2). Data are expressed as the mean ± SD. *Y*‐axis is the normalized ATP level relative to DMSO. (I) EGF ligand dependency in accordance with gefitinib sensitivity. Three gefitinib‐sensitive organoids (033‐, 034‐, and B018‐O) and two nonsensitive organoids (032‐ and 023‐O) displayed different growths when EGF was withdrawn from the culture medium. Data are expressed as the mean ± SD (*n* = 3). *Y*‐axis is the normalized ATP level relative to control medium containing EGF. *P*‐values were estimated using the Mann–Whitney test, **P‐val < 0.01. PDOs, patient‐derived organoids.

Not every patient possessed a druggable mutation such as patients 023 or 032. Additionally, even if tumors possess a druggable mutation, efficacy may not be observed, such as the case of 030‐O, in which mutation‐based drug treatment failed to induce regression. For these cases of resistance to standard therapeutics, we conducted drug screenings with nontraditional therapeutics. Concerning drug repurposing, organoids were screened using a library of FDA‐approved oncology drugs. To mimic patients with poor responses to standard‐of‐care treatment, drug screening was performed using 10 PDOs with an organoid score of 2.5 or higher that were obtained from patients with disease progression (Fig. S7A). Drugs were dispensed using an automated laboratory workstation. To verify that the screening was successfully conducted, we compared the replicated screening results and confirmed that each replicated screening produced similar results based on the AUC (median Pearson’s *r* = 0.978, Fig. 6A–F). In the drug library, there were a few drugs that shared target molecules, such as CDK4/6 (ribociclib, palbociclib, and abemaciclib) or PARP (niraparib, talazoparib, rucaparib, and olaparib). Therefore, we also confirmed whether drugs with identical targets had similar anticancer effects. We observed relatively similar responses to drugs that shared targets in various organoids (Fig. 6G, Fig. S7B).

More specifically, there were eight EGFR/HER2‐targeted agents in the FDA‐approved oncology drug library. Gefitinib is a therapeutic option for EGFR pathway‐addicted advanced non‐small‐cell lung cancer (NSCLC) [41, 42]. Among our PDOs, 033‐O, 034‐O, and B018‐O were sensitive to gefitinib relative to 023‐O and 032‐O despite the absence of EGFR mutations (Fig. 6H). To validate the different responses to gefitinib, we cultured PDOs in culture medium without EGF. As a result, three PDOs that were sensitive to gefitinib exhibited reduced organoid growth when cultured in EGF‐free medium. Conversely to this and similar to the responses to gefitinib, no significant changes were observed in 023‐O and 032‐O when cultured with or without EGF (Fig. 6I, Fig. S8). The different drug responses and EGF dependency of PDOs indicated that EGFR‐targeted therapy might be one of the possible options to consider for patients 033, 034, and B018.

## 4 Discussion

Conventional chemotherapeutic agents and mutation‐based targeted therapies do not always lead to favorable patient responses [43, 44]. This indicates that, despite its importance, mutational profiling alone is insufficient for selecting therapeutic strategies. Indeed, patients with similar mutation profiles can have differential drug responses, emphasizing the need for a more personalized model of care. In this aspect, PDOs are expected to emerge as a model that provides an effective and reproducible translational system, to overcome the limitations of previous cancer models [45]. The major challenge for personalized model platforms is whether PDOs maintain the original pathology and genetic features of the patient and display a similar response to treatment. There are fabulous studies that showed the possibility of PDOs as patient mimic model system [46, 47]. To get additional insight of PDO, here, we established organoids from patients who had not been treated any form of anticancer treatment before sample collections. We introduced a scoring system called the organoid score to reflect the anticancer therapy regimens received by matched patients and compared the organoid score with responses in patients. Using this metric, we found correlations between the organoid score and change of the tumor burden after standard‐of‐care therapy. We also confirmed that the organoid score is a marker for disease progression through the Kaplan–Meier analysis. Moreover, using therapy‐refractory organoids with high organoid scores, we proposed a second treatment option through drug screening using clinical trial and FDA‐approved drugs. These results indicated that PDOs and the organoid score comprise a preclinical model in the aspects of therapy prediction and recommendations for personalized therapy.

Despite the predictive ability of the organoid score, it cannot be applied for all therapies in PDOs. Bevacizumab is prescribed to treat metastatic CRC [48]. However, we could not include bevacizumab in the organoid score because its main anticancer effect is antivascularization, which cannot be evaluated using the organoid platform [2, 49]. To overcome this hurdle, organoid‐based coculture or assembloid study of CRC would be helpful [50]. Additionally, a larger cohort study will be required to validate the reliability of the organoid score. Regardless, our results support organoids as a potentially powerful preclinical model. There was one sample (049‐O) for which no agreement between the organoid and tumor tissue was observed. We analyzed WES data to determine the possibility of sample cross‐contamination. However, identical sequences were in perfect alignment with PDOs and tissue. To avoid similar issues in the future, we propose the use of multiple sections of tissue fragments from each patient to account for intra‐tumor heterogeneity [20].

One of the key features of advanced tumors is metastasis. Developing organoids with invasion properties [51] or producing organoids from metastatic tumor tissue is also possible [52, 53], as demonstrated for the patient 046 ascites‐derived tumor organoid. Therefore, sequential integrative analysis of normal, primary, and metastatic tumor organoids might represent a promising approach to accurately capture tumor heterogeneity and progression.

We identified two groups of PDOs with contrasting responses to EGFR‐targeting drugs and EGF ligands despite the absence of oncogenic mutations in RAS and PIK3CA (Fig. 6H,I). Of note, 033‐O and 034‐O (sensitive to EGF ligand and EGFR‐targeted drugs) had approximately twofold higher *EGFR* and *ERBB2* RNA expression than 032‐O (nonsensitive PDO), but B018‐O and 023‐O exhibited relatively similar RNA expression for these genes (data not shown). Therefore, further research on the difference in the response mechanism between B018‐O and 023‐O would be helpful for applying gefitinib as an off‐label drug in CRC.

Of the 54 patients, 17 patients were evaluated as disease progression after first therapy. Among them, six patients were assessed for the response to second‐line therapy. In two patients, the best response to second‐line therapy was PD, and in the other four patients, the best response was SD. Interestingly, we confirmed that the average drug score of two patients with PD (score ≥ 3.25) was higher than that of the four patients with SD (score ≤ 3; Tables S1 and S3). The use of chemotherapy‐naïve organoids for predicting the response to second‐line therapy would require a careful approach. However, PDOs are expected as a test platform for identifying multi‐drug‐resistant tumors, followed by the rapid application of alternative therapy to achieve a better prognosis. Recently, a study using genome‐wide CRISPR library screening of normal human intestine organoids was conducted [54, 55]. Additionally, 3D environment‐specific vulnerabilities were explored through cancer spheroid culture [56]. These studies suggested that 3D organoid‐based studies of drug response mechanisms could be conducted by combining drug and CRISPR library screening. Furthermore, research on this concept will enable a deeper understanding of cancer, which has not been adequately represented by *in vitro* 2D culture, to bridge the gap between *in vivo* and *in vitro* investigations.

## 5 Conclusions

Patient‐derived organoids successfully recapitulated the genetic, phenotypic, and varying drug responses of matched patients with CRC. Moreover, we demonstrated the potential utility of the organoid score system based on drug response/nonresponse measurement in organoid viability assays. On the basis of these findings, this organoid model may provide a foundation for a highly valuable preclinical *ex vivo* model to predict patient responses to anticancer drugs. Furthermore, this PDO system can be used to suggest alternate or off‐label therapeutic options for patients who do not respond to traditional therapeutic regimens.

## Conflict of interest

The authors declare no conflict of interest.

## Author contributions

YWC, DWM, HPK, SHS, SWH, and TYK contributed to the conception and design of this study. YWC, DWM, and JY contributed to organoid experiments and data collections. YA and YSJ contributed to analyzing sequencing data. SK, JC, JPI, and KJP contributed to patient’s tissues and data collection.

### Peer Review

The peer review history for this article is available at https://publons.com/publon/10.1002/1878‐0261.13144.

## Supporting information


**Fig. S1.** Overall Workflow of this study.

**Fig. S2.** Establishment and functional validation of adjacent colon mucosa‐derived normal organoids.

**Fig. S3.** Mixed morphology of cystic/round and aggregated forms in case of 032‐O.

**Fig. S4.** Homozygous large deletion in 023 (*RNF43*) and 032 (*TP53*) patient‐derived organoids and matched tissues.

**Fig. S5.** Results of NGSCheckMate software.

**Fig. S6.** Highly sensitive response to treatment with porcupine inhibitors in *RNF43‐*mutant 023‐O and 052‐O.

**Fig. S7.** FDA‐approved 57‐drug library screening using 10 chemotherapy‐refractory patient‐derived organoids.

**Fig. S8.** Representative images of tumor organoid growth with or without EGF in the culture medium.

**Table S1.** Cohort information in this study.

**Table S2.** Patient‐derived tumor organoid mutation profile.

**Table S3.** Patient‐derived tumor organoid drug response(a) and organoid score(b).

**Table S4.** Colorectal cancer related pathway gene mutation in LGK974 sensitive organoid.

Supplementary Material

## Data Availability

The data that support the findings of this study are available from the corresponding author upon reasonable request.
