# Supplementary figures and images for: Patient‐derived organoids as a preclinical platform for precision medicine in colorectal cancer
Source: Mol Oncol. 2022 Jan 1;16(12):2396–412. doi: 10.1002/1878-0261.13144 (PMC9208081; doi:10.1002/1878-0261.13144)

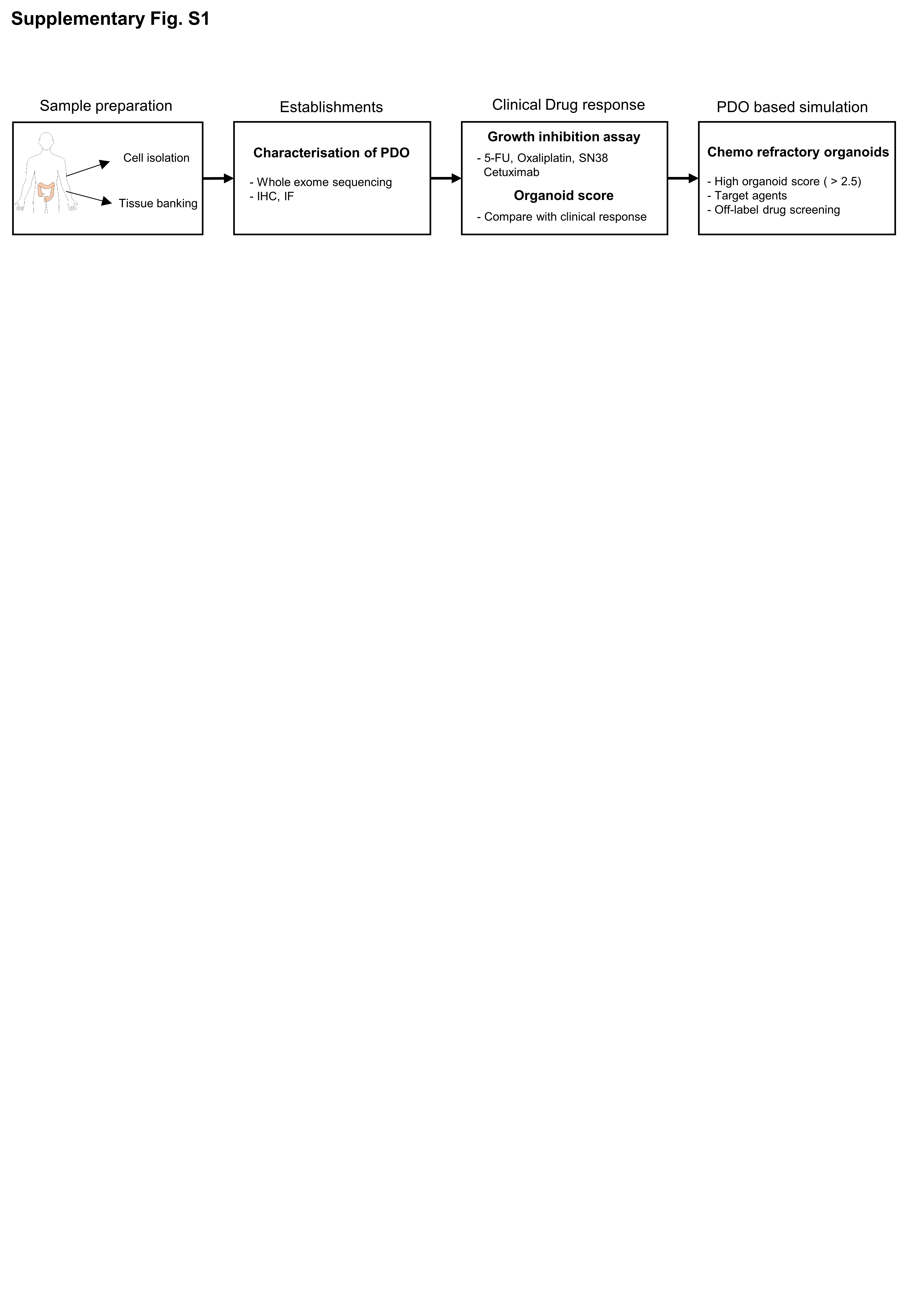

Supplement: Supplementary file 1 — Fig. S1. Overall Workflow of this study. [file MOL2-16-2396-s012.TIF]

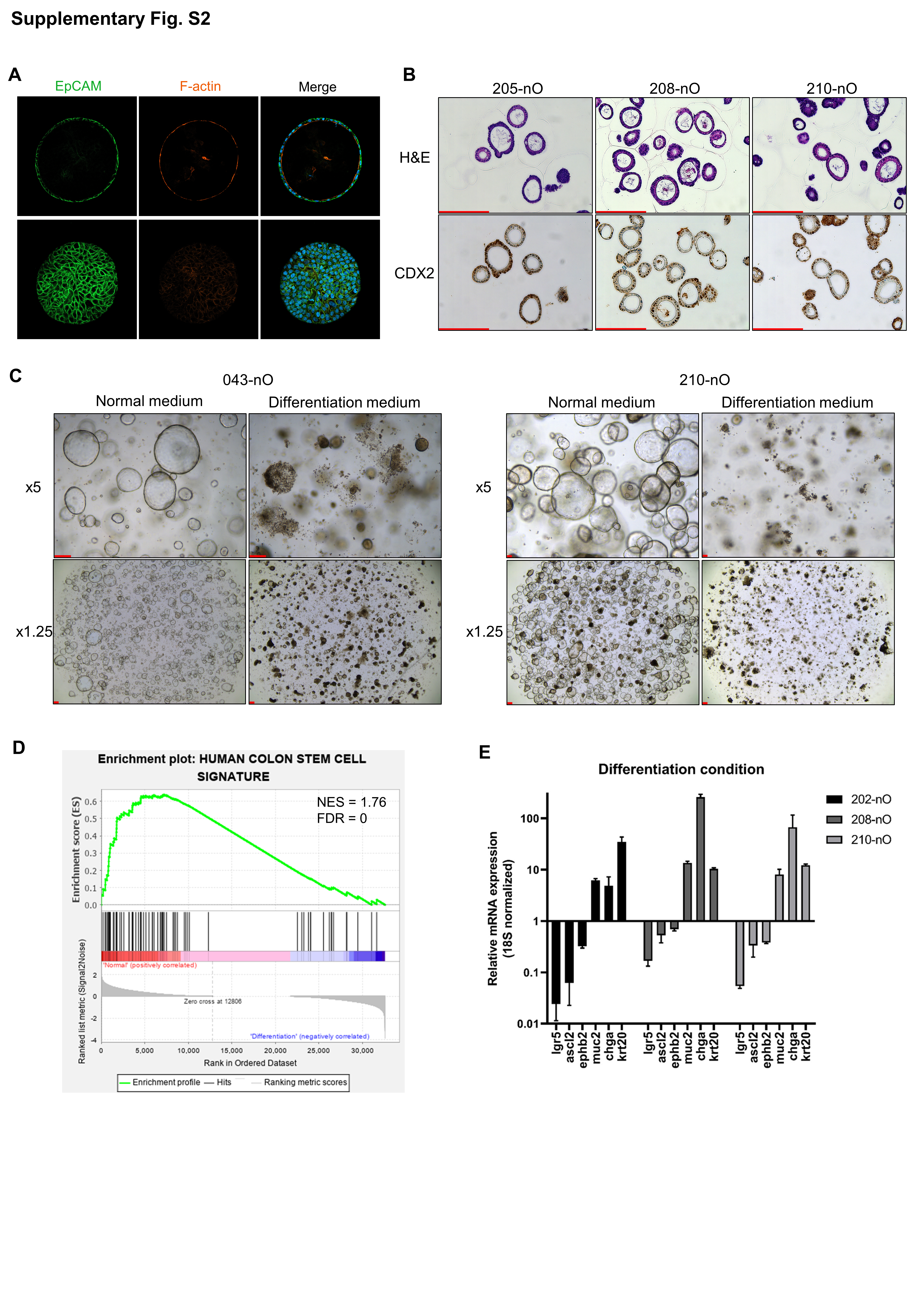

Supplement: Supplementary file 2 — Fig. S2. Establishment and functional validation of adjacent colon mucosa‐derived normal organoids. [file MOL2-16-2396-s008.TIF]

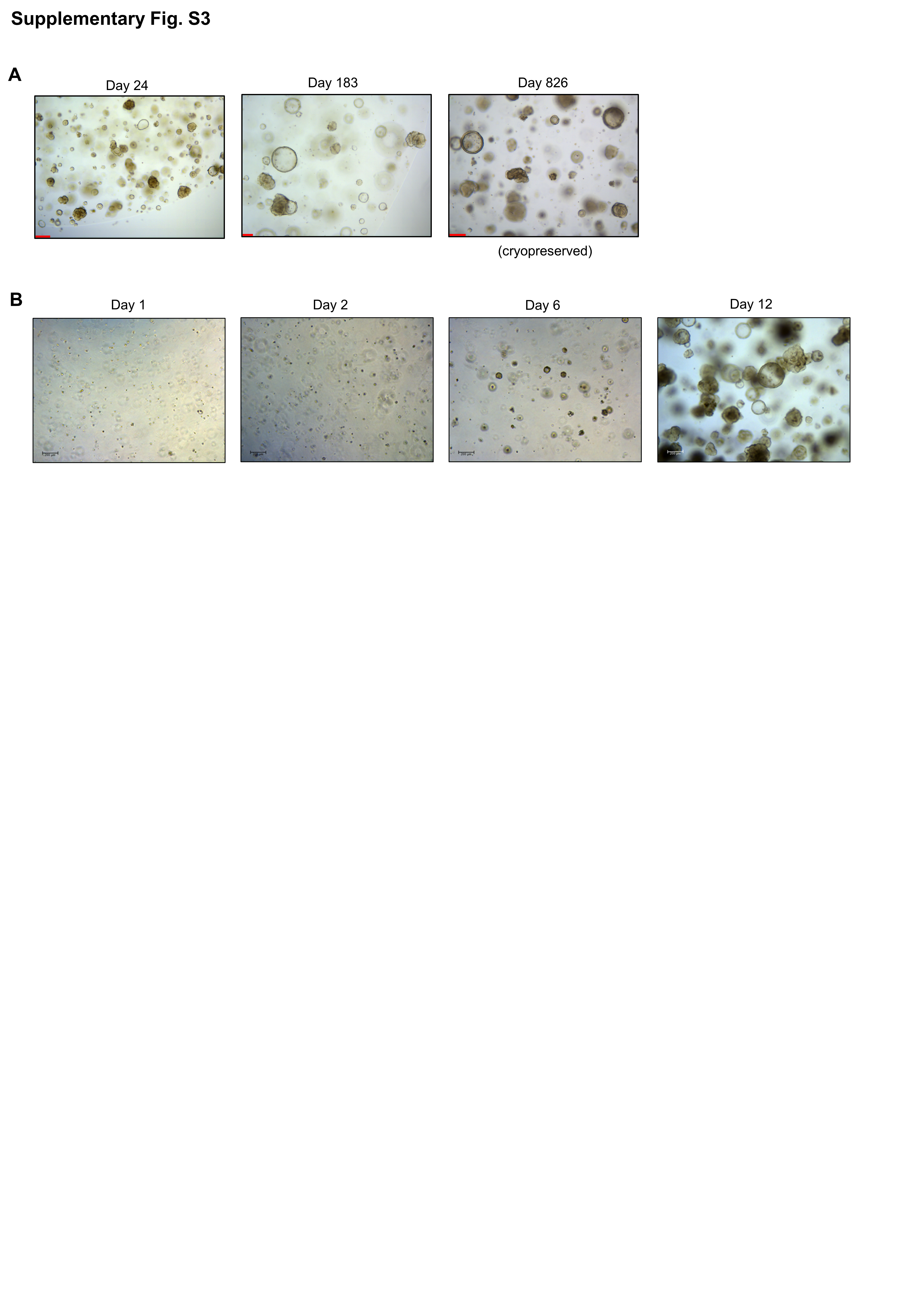

Supplement: Supplementary file 3 — Fig. S3. Mixed morphology of cystic/round and aggregated forms in case of 032‐O. [file MOL2-16-2396-s006.TIF]

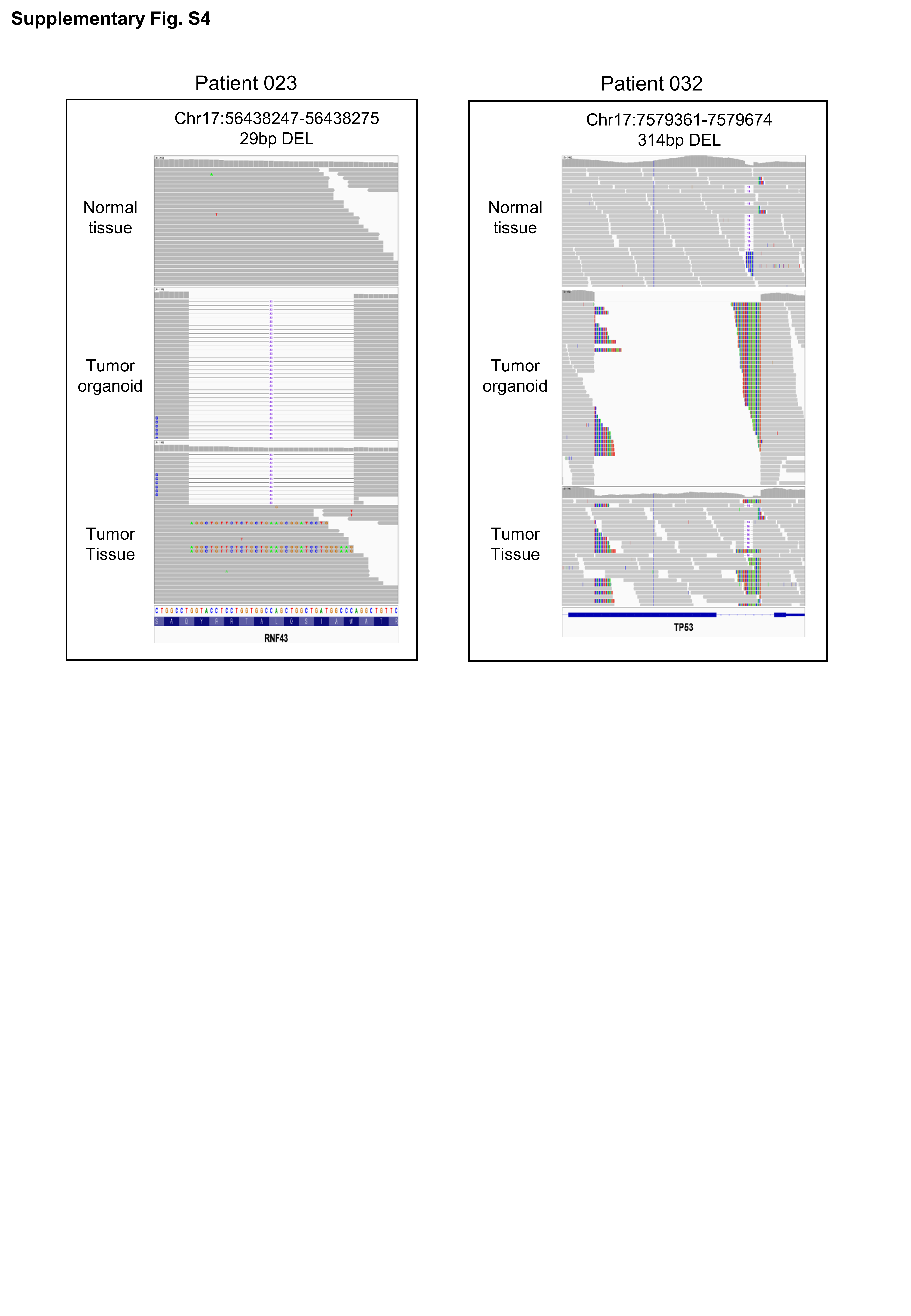

Supplement: Supplementary file 4 — Fig. S4. Homozygous large deletion in 023 (RNF43) and 032 (TP53) patient‐derived organoids and matched tissues. [file MOL2-16-2396-s009.TIF]

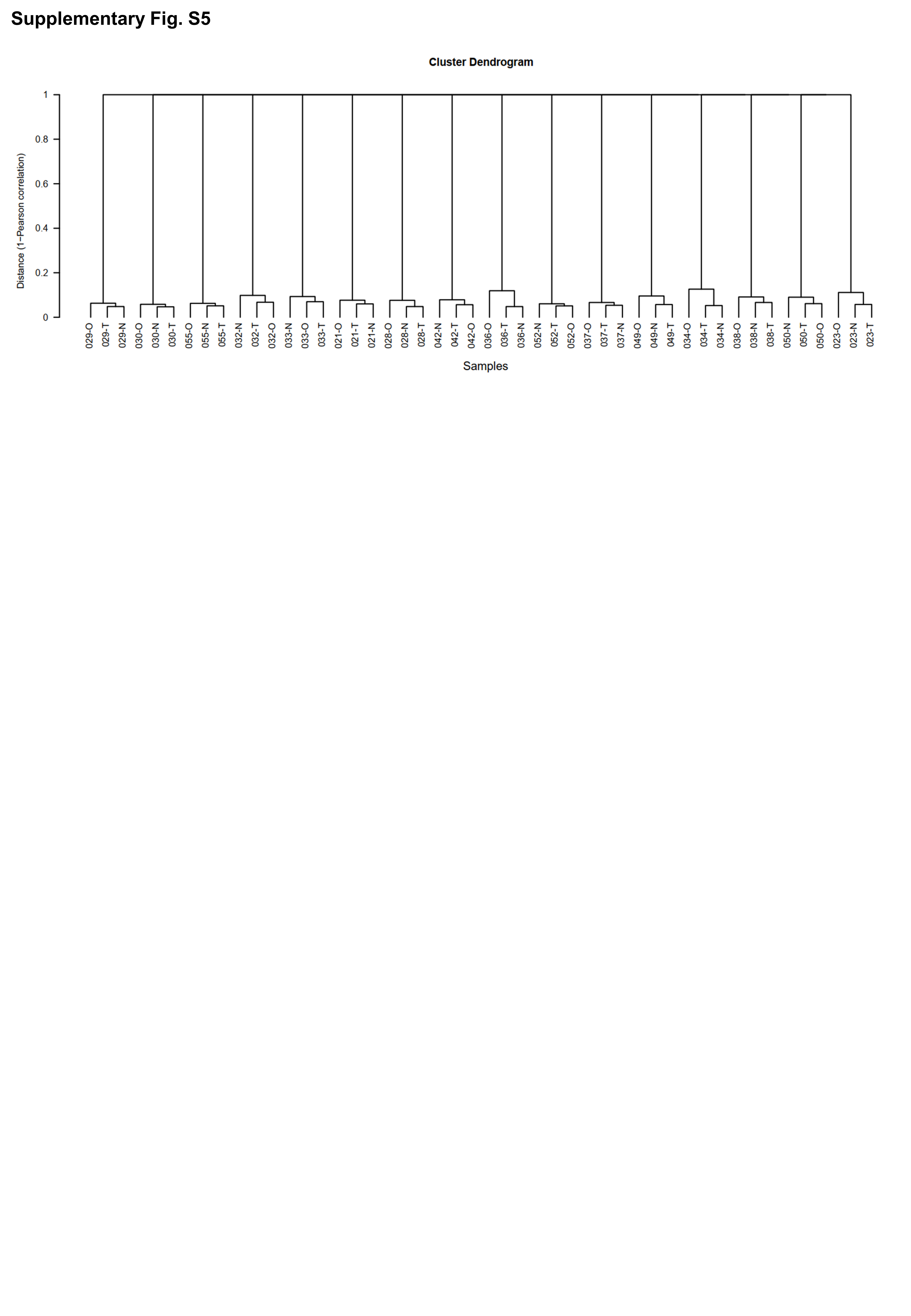

Supplement: Supplementary file 5 — Fig. S5. Results of NGSCheckMate software. [file MOL2-16-2396-s004.TIF]

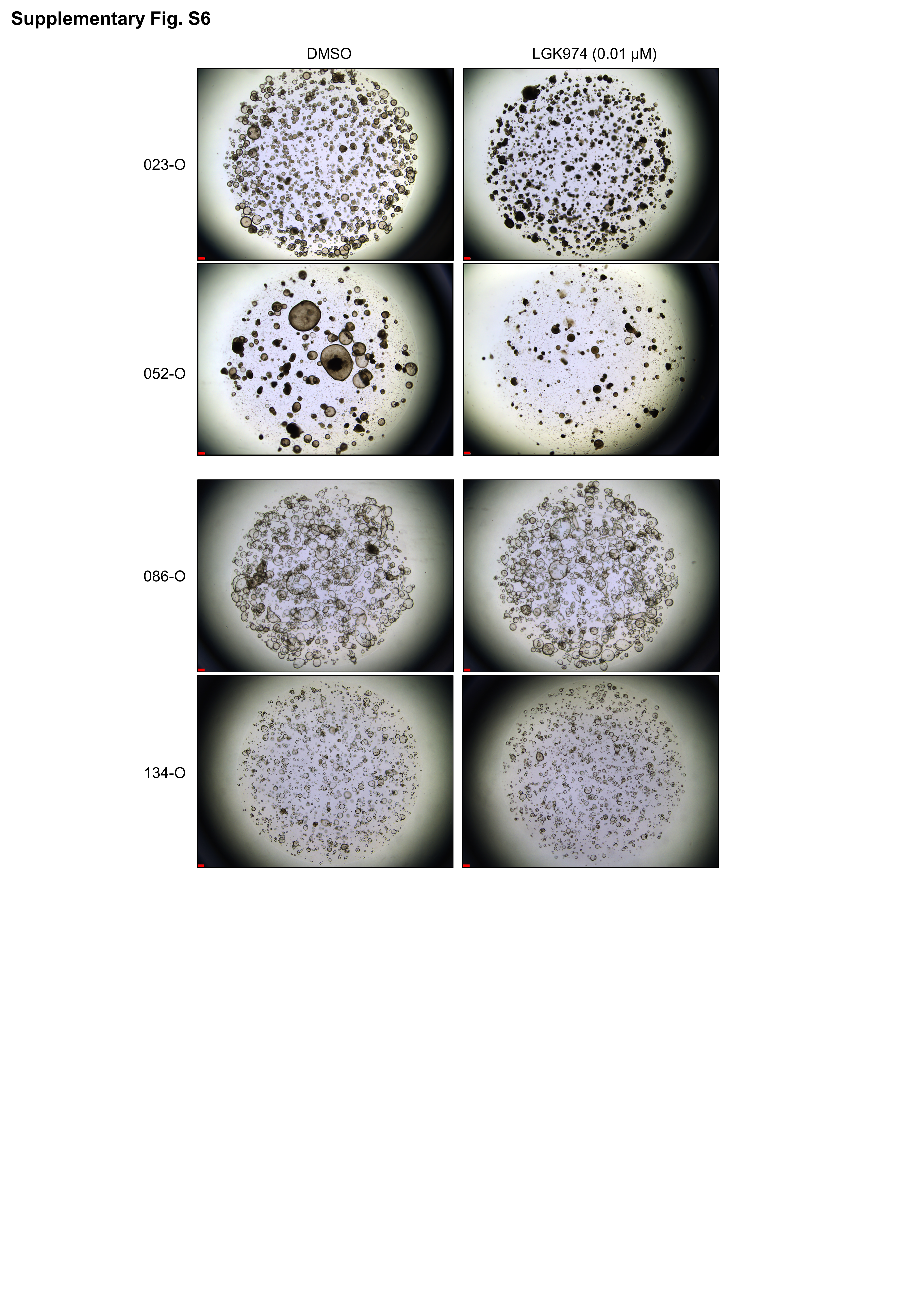

Supplement: Supplementary file 6 — Fig. S6. Highly sensitive response to treatment with porcupine inhibitors in RNF43‐mutant 023‐O and 052‐O. [file MOL2-16-2396-s003.TIF]

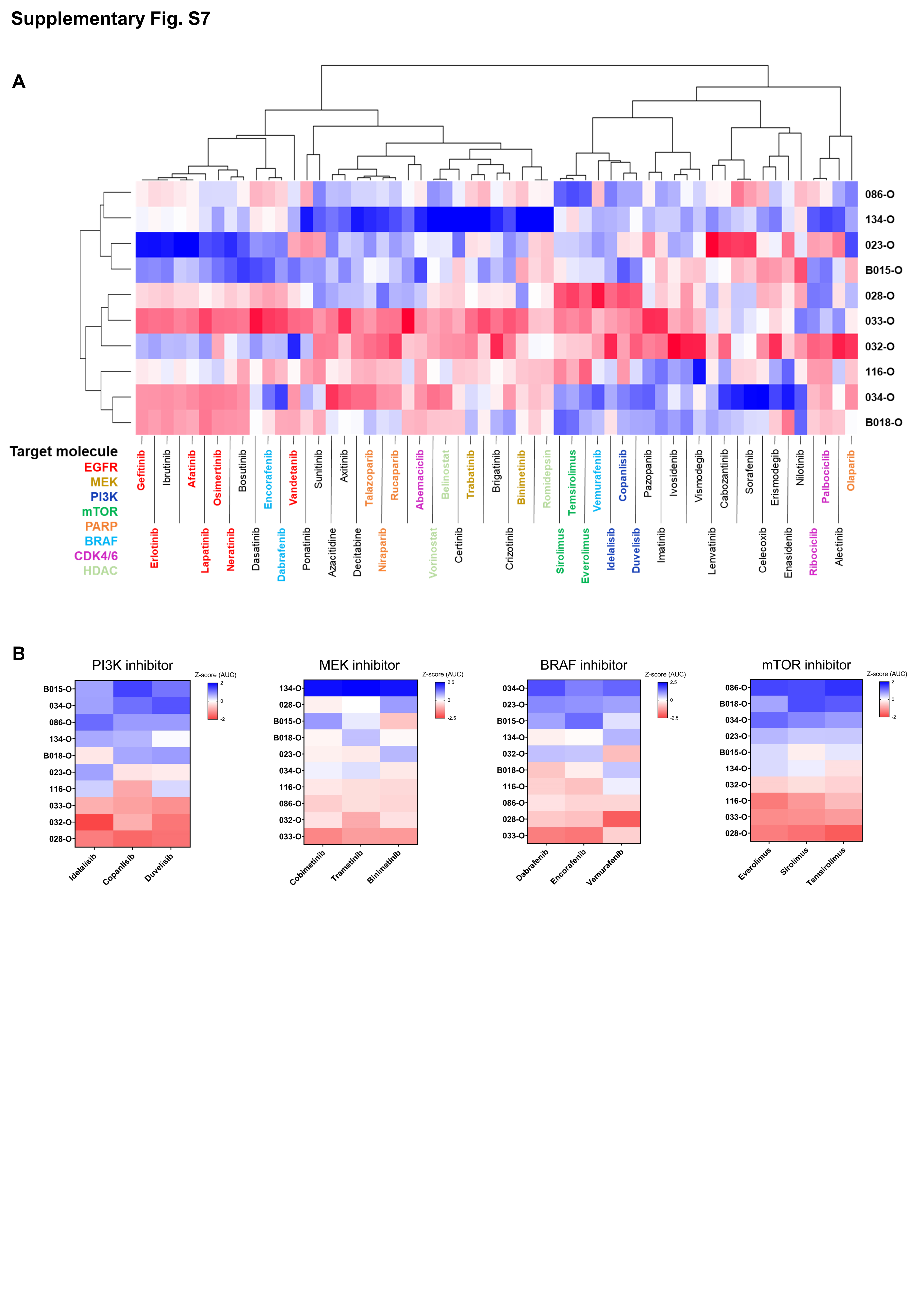

Supplement: Supplementary file 7 — Fig. S7. FDA‐approved 57‐drug library screening using 10 chemotherapy‐refractory patient‐derived organoids. [file MOL2-16-2396-s001.TIF]

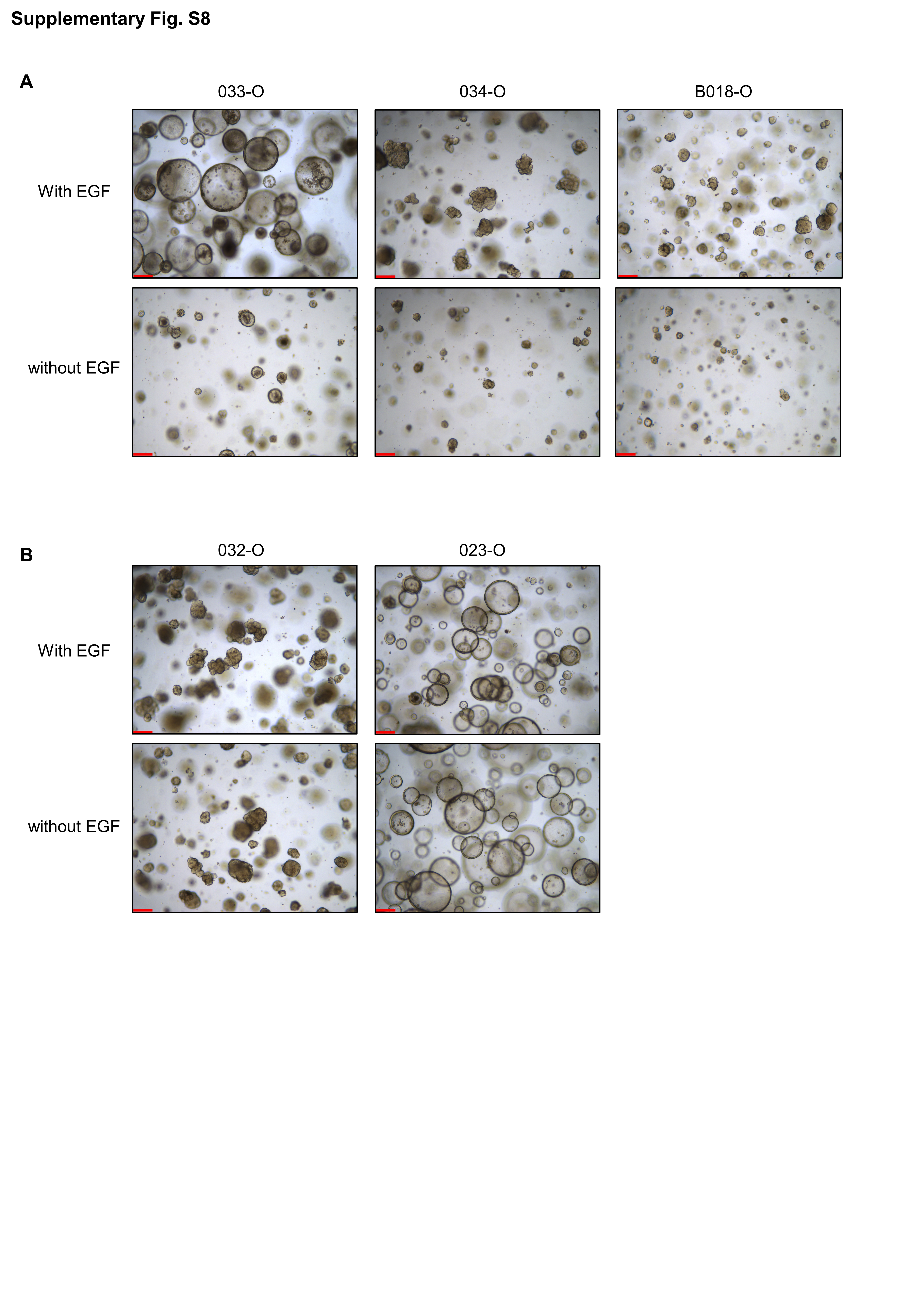

Supplement: Supplementary file 8 — Fig. S8. Representative images of tumor organoid growth with or without EGF in the culture medium. [file MOL2-16-2396-s007.TIF]
