# Supplementary material for: Patient‐derived organoids as a preclinical platform for precision medicine in colorectal cancer
Source: Mol Oncol. 2022 Jan 1;16(12):2396–412. doi: 10.1002/1878-0261.13144 (PMC9208081; doi:10.1002/1878-0261.13144)
Supplement: Supplementary file 13 — Supplementary Material [file MOL2-16-2396-s010.docx]

**Supporting Information**

**Supplementary figure legends**

**Supplementary Fig. S1** Overall Workflow of this study. PDOs were established with a 75% success rate. PDOs were characterized by morphological features and mutations. Then, PDOs were screened for sensitivity to clinical level drug and FDA-approved drug library. PDO, patient-derived organoid.

**Supplementary Fig. S2** Establishment and functional validation of adjacent colon mucosa-derived normal organoids. (A) Representative immunofluorescence images of a colon normal organoid composed of EpCAM-positive epithelial cells. Upper and lower images represent the same organoid with different foci. (B) Representative immunohistochemical images of CDX2-positive colon normal organoids. CDX2 was used for colon epithelial marker. (C) Structure disruption and growth suppression of a colon normal organoid in Wnt3a-depleted medium. Images were obtained after 7 days of incubation in differentiation medium. (D) Gene set enrichment analysis of RNA sequencing data. Each normal organoid was cultured with or without Wnt3a conditioned medium (n = 3, each condition). (E) Relative mRNA expression change (quantitative RT-PCR) after normal organoid differentiation. Data are expressed as the mean ± SD (n = 3). RNA samples for RNA sequencing and quantitative RT-PCR were collected after incubation for 4 days in each medium medium. H&E, hematoxylin and eosin.

**Supplementary Fig. S3** Mixed morphology of cystic/round and aggregated forms in case of 032-O. (A) Mixed cystic/round and aggregated morpholgies were maintained after 6 months of culture and cryoperservation. The day was counted from the date of primary culture. (B) Organoid formation after single-cell dissociation. Dissociated single cells also formed organoid with mixed morphologies. The day was counted from the date after single cell dissocation and reseeded in Matrigel.

**Supplementary Fig. S4** Homozygous large deletion in 023 (*RNF43*) and 032 (*TP53*) patient-derived organoids and matched tissues identified via whole exome sequencing. Images were captured from Intergrative Genomics Viewer of normal tissues, tumor tissues, and tumor organoids.

**Supplementary Fig. S5** Results of NGSCheckMate software to validate sample identity using whole exome sequencing data indicating the absence of mismatched samples.

**Supplementary Fig. S6** *RNF43-*mutant 023-O and 052-O were highly sensitive to treatment with porcupine inhibitors compared with *RNF43* wild-type organoid 086-O and 134-O. The images were obtained 6 days after treatment. Scale bar = 200 µm.

**Supplementary Fig. S7** FDA-approved 57-drug library screening using 10 chemotherapy-refractory patient-derived organoids. (A) Heat-map of drug screening results with unsupervised clustering using the Z-score. The Z-score was calculated using the area under the drug response curve, indicating the relative treatment efficiency. Each color in the drug name indicates the target molecule. (B) Molecular targeted drugs that shared the same target showed similar response pattern in 10 PDOs. PDO, patient-derived organoid..

**Supplementary Fig. S8** Representative images of tumor organoid growth with or without EGF in the culture medium. (A, B) EGFR-targeted drug sensitive group (A) exhibited EGF ligand dependency and growth depletion compared with EGFR-targeted drug non-sensitive group (B). Both groups had no oncogenic mutations in KRAS, NRAS, BRAF and PIK3CA.
